# Sustainability of One WASH Facilities in the Rural Settings of North Shoa Zone, Amhara Region, North East Ethiopia, 2020

**DOI:** 10.1155/2022/1711389

**Published:** 2022-02-14

**Authors:** Muluken Tessama Aemiro, Yohannes Getachew

**Affiliations:** ^1^Department of Public Health, Asrat Woldeyes Health Science Campus, Debre Berhan University, P. O. Box 445, Debre Berhan, Ethiopia; ^2^Amhara Regional Health Bureau, North Shoa Zone Health Department, Debre Berhan, Ethiopia

## Abstract

**Background:**

Water, sanitation, and hygiene (WASH) is considered as one term and recognizes that the three are closely related. The Government of Ethiopia launched the programme to improve the way water, and sanitation is provided to the people improving the WASH financing effectively, decreasing school children drop-out rates and improving the health status. The main aim of this study is to assess factors related to the sustainability of one WASH facilities in the rural settings of North Shoa zone, North East Ethipia.

**Methods:**

Institutional- and community-based cross-sectional study was conducted. Taking the total number of woredas in the zone, 20 of them were rural project woredas during the first phase of the program and clustered in 6 subzones, and 6 woredas had been selected randomly by the lottery method from each subzones. A total of 768 households were randomly selected based on the proportional size of the number of households in each woreda. A structured questionnaire was used for this study. The data were collected via interview. Multivariable logistic regression analysis was used to identify independent predictors with *P* < 0.05 and confidence interval of 95% considering statistically significant.

**Result:**

Among a total of 768 HHs intended to be involved, 689 were involved with 90% response rate. The communities WASH facilities were assessed to be unsustainable by more than half of the respondents (372 (54 percent)) HHs replies. Distance from current water source, community participation during water construction, practice of CLTSH in the village, declaration of open defecation free (ODF) in the village, and existence of health institution near the village were found positively associated with sustainability of one WASH facilities.

**Conclusion:**

The sustainability of WASH facilities was revealed to be relatively low. Considering distance from water source, community participation, practice of CLTSH in the village, village declared ODF, and existence of health institution are mandatory to all stake holders participating in WASH activities before, during, and after the implementation of the project.

## 1. Background

Water, sanitation, and hygiene (WASH) is considered as one term and recognizes that the three are closely related. The supply of water free from any form of disease-causing agents is considered as safe water supply. According to WHO, 20 liters of water per person per day is adequate water supply that satisfies the minimum amount safe physical reach from home or cleanliness of the face, hair, body, feet, and clothing, and for women and girls, menstrual hygiene is considered as accessible water supply [1].

WASH within 1 km or a 30-minute round trip has significant potential to improve institution, based on international development agencies [2].

The provision of facilities and services for the safe disposal of human urine and feces is considered as sanitation. A set of practices for the preservation of health and healthy living refers to hygiene. The most important element is hand washing with soap or ash, personal health, life expectancy, student learning, gender equality, and other important issues of international development [3].

According to the United Nation's MDGs, upgrading of WASH services in Target 7.C: “Halve, by 2015, the proportion of the population without sustainable access to safe drinking water and basic sanitation [4],” has been replaced by the SDGs where Target 6 includes “assurance availability and sustainable management of water and sanitation for all” [5].

The administration of Ethiopia launched the WASH program to reduce school children dropout rate, making financing for WASH more effective and improving the health status, seven years covering the period from July 2013 to June 2015 for Phase I and from July 2015 to June 2020 for Phase II [6].

Government and donors jointly have accepted the need for greater coordination, and to achieve this, we have agreed to move from “a project to a program approach” [7].

WASH United at this time works in 8 countries in Sub-Saharan Africa and in India. The countries in Africa are Burkina Faso, Ethiopia, Ghana, Kenya, Lesotho, Mali, Tanzania, and Uganda [8].

In Ethiopia, the early September 2013 One WASH National Program (OWNP) was launched based on a sector-wide sanitation and water approach (SWAP) and includes ministries of Water, Health, Education and Finance and the principal development partners with an implementation period of seven years covering the period from July 2013 to June 2015 for Phase I and from July 2015 to June 2020 for Phase II, in activities of four components: Rural and Pastoral WASH, Urban WASH, Institutional WASH and Program Management, and Capacity Building [9].

The program implemented over a period of five years starting in July 2014 and ended in May 2019. It was implemented in all the 9 regions and the two city administrations of Ethiopia [7].

Lack of access to sanitation, use of unsafe drinking water, and poor hygiene together are responsible for about 88% of all deaths from diarrheal diseases in developing countries. Regionally, it is being implemented in 89 woredas and 41 towns as of rural and urban sanitation program. Twenty-two woredas and seven towns are the one WASH supported woredas and towns from Northern Shoa administrative zone [10].

### 1.1. Study Objective

The objective of this study was to assess factors related to sustainability of one WASH facilities in the rural settings of North Shoa zone, Amhara Region, North East Ethiopia, 2020.

## 2. Methods

### 2.1. Study Area

The study was conducted in North shoa zone, which is found in Amhara regional state. North shoa zone is one of the zones in Amhara National Regional State.

### 2.2. Study Design and Period

An institution- and community-based cross-sectional study was conducted from the 15th of November to the 30th of December 2020 in North shoa zone, Amhara National Regional State.

### 2.3. Source Population

All households in the project areas of 20 rural woredas of North Shoa.

### 2.4. Study Population

All selected households from the 6 randomly selected woredas. Respondents in the project rural woredas were involved in the study. During the data collection period, households not open were excluded from the study.

The sample size for this study was determined using a single population proportion formula, where *N* = size of the study population, *n* = sample size, *p* = sustainability to WASH facility to 50%, *d* = desired level/margin of error (5%), *z* = standard normal distribution curve value for the 95% confidence interval (1.96), and design effect = 2. Based on the formula, the sample size was calculated to be 768 respondents.

### 2.5. Data Collection Procedure

A structured interview questionnaire was used to collect the data. We also pretested the instrument and found that it is appropriate in the study area. Causes that could possibly affect the sustainability to WASH facility were selected from the literature. For the consistency of the questionnaire, the quantitative data were first prepared in English, translated to Amharic, local language, and then translated back to English by language professionals. The questionnaire was pretested on 5% of study participants. Six trained Environmental Health Technicians were assigned to collect the data. Supervisors monitor the entire data collection process.

### 2.6. Data Quality Management

In order to ensure the quality of the data, structured and pretested questionnaires were used to collect data. Training was given by the principal investigator on the objective, relevance of the study, confidentiality of information, respondent's right, informed consent and techniques of interview with respect to the study.

### 2.7. Data Processing and Analysis

The data were cleaned, entered, and coded using EPI info version 3.5.1 and exported to SPSS version 20 for analysis. Frequencies and cross-tabulations were used to summarize the data. Bivariate and multivariate analyses were executed to test associations of factors associated with the sustainability to WASH facility with the other covariates. A *P* value of ≤0.05 was considered as significantly associated with sustainability to WASH facility.

## 3. Results

### 3.1. Sociodemographic Characteristics

A total of 768 households were supposed to be included in the study. From these total number of HHs which had been visited during the study, 689 HHs were involved in the study with a response rate of 90%. Participants were from 17 to 75 years with a mean age of 37 SD ± 10.7 years. Out of 689 household heads, 393 were females and 515 (74.7%) were single.

### 3.2. Sustainability of One WASH Facilities in the Rural Settings of North Shoa Zone, Amhara Region, Ethiopia

Distance from the current water source, community participation during water construction, practice of CLTSH/ignition done in the village, village/Kebele declared ODF, and existence of health institution in/near the village were found to be positively associated with the sustainability of one WASH facilities.

**Figure 1:**
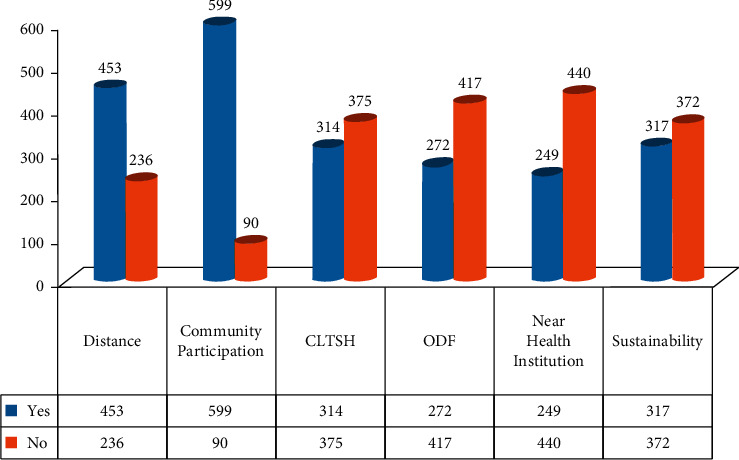
Factors related to sustainability of one WASH facilities in the rural settings of North Shoa, Ethiopia,2020.

Among 689 household heads, 317 (46%) HHs responses show that the communities' WASH facilities were sustainable and 372 (54%) HHs responses show that the communities' WASH facilities were found to be not sustainable to WASH facilities (Figure 1).

Concerning the distance from the current water source, 453 (65.7%) of HHs were found at a distance <1.5 km and 236 (34.3%) of HHs were found at a distance >1.5 km (Figure 1).

From the community participation point of view, 599 (86.9%) HHs participated in labour, money, kind, or any form of participation during water source construction and 90 (13.1%) HHs did not participate (Figure 1).

A total number 314 (45.5%) of HHs responded that CLTSH was practiced in their village and 375 (54.5%) HHs responded that CLTSH was not practiced in their village (Figure 1).

Regarding open defecation free, 272 (39.4%) HHs responded that their village had declared ODF and 417 (60.6%) HHs responded that their village had not declared ODF (Figure 1), and concerning the existence of health institution near the village, 440 (63.8%) of HHs had health institutions near their village whereas 249(36.2%) of HHs had no health institutions in/near their village (Figure 1).

### 3.3. Factors Associated with Sustainability of One WASH Facilities Using Bivariate Logistic Regression Analysis

Distance from the current water source, community participation during water construction, practice of CLTSH/ignition done in the village, whether the village declared ODF or not, and the existence of health institution in/near the village were found to be positively associated with sustainability of one WASH facilities.

### 3.4. Distance from the Current Water Source

WASH facilities found within a distance of ≤1.5 km are 4.36 times more likely to be sustainable than those which are >1.5 km and apart (COR = 4.36, 95% CI: 3.07–6.22) (Table 1).

### 3.5. Community Participation during Water Source Construction

WASH facilities at which community was participated during their construction are 23.53 times more likely to be sustainable than those at which community was not participated during their construction (COR = 23.53, 95% CI: 5.25–64.94) (Table 1).

### 3.6. Practice of CLTSH

WASH facilities in areas where CLTSH was practiced were 3.48 times more likely to be sustainable than those areas where CLTSH was not practiced (COR = 3.48, 95% CI: 2.54–4.77) (Table 1).

### 3.7. Whether the Village Had Declared ODF or Not

WASH facilities in areas where ODF was declared are 1.38 times more likely to be sustainable than those found in areas where ODF was not declared (COR = 1.38, 95% CI: 1.38–2.52) (Table 1)

### 3.8. The Existence of Health Institution in/near the Village

In areas where there were health institutions near the village, WASH facilities were found to be more sustainable 7 times than those where there were no health institutions near the village (COR = 7.43, 95% CI: 5.21–10.59) (Table 1).

### 3.9. Factors Associated with Sustainability of One WASH Facilities Using Multivariate Logistic Regression Analysis

Distance from the current water source, community participation during water construction, practice of CLTSH/ignition done in the village, whether the village declared ODF or not, and the existence of health institution near the village were found to be positively associated with the sustainability of one WASH facilities.

### 3.10. Distance from the Current Water Source

WASH facilities found within a distance of ≤1.5 km are 6 times more likely to be sustainable than those which are >1.5 km and apart (AOR = 6.46, 95% CI: 4.11–10.14) (Table 1).

### 3.11. Community Participation during Water Source Construction

WASH facilities at which community has participated during their construction are 29 times more likely to be sustainable than those at which community has not participated during their construction (AOR = 29.43, 95% CI: 9.49–91.29) (Table 1)

### 3.12. Practice of CLTSH

WASH facilities in areas where CLTSH was practiced were 3.47 times more likely to be sustainable than those found in areas where CLTSH was not practiced (AOR = 3.47, 95% CI: 2.29–5.24) (Table 1).

### 3.13. Whether the Village Had Declared ODF or Not

WASH facilities in areas where ODF was declared are 1.62 times more likely to be sustainable than those found in areas where ODF was not declared (AOR = 1.62, 95% CI: 1.1–2.52) (Table 1).

### 3.14. The Existence of Health Institution near the Village

In areas where there were health institutions near the village, WASH facilities were found to be more sustainable 8 times than those where there were no health institutions near the village (AOR = 8.08, 95% CI: 4.75–13.74) (Table 1).

## 4. Discussion

This study has obtained important information in assessing regarding to the sustainability of one WASH facilities and associated factors among households in the rural settings of North Shoa, Ethiopia.

One WASH National Program is the umbrella program for all water-, sanitation-, and hygiene-related hardware and software activities in Ethiopia, and it is also one of the main national instruments for achieving goals and targets of the government's GTP plan [11–14].

As mentioned in statement of problem, the main thing which has been challenging and hindering the effort in water supply and sanitation sector is the lack of sustainability and increase of nonfunctioning schemes in significant number among any number of implemented schemes.

According to this study, 317 (46%) of respondents of HHs had sustainable WASH facilities in their settings. In a study conducted in the northern Gondar rural setting, 86.6% of the community's participation and 78% of the HHs being in a distance <1.5 km of the facilities contribute to the functionality of the facilities. In this study, only 36% of the total studied HHs were within the radius of water source. The same type of study in Northern Gondar in 2002 showed only 18.8% was in the context [15].

Both studies also showed that distance is a strongly associated factor with OR = 6.46 (4.11–10.14), 95% CI OR = 5.57, respectively.

Of the total respondents, 87% had contributed to either collecting money or kind to the water and sanitation project development. The observed results of the participation level are higher than those of the estimated fifty percent of the community. Therefore, we could say that participation of the households possibly had a positive association with the sustainability of water and sanitation projects. This result revealed that communities understand reasons for their participation: aimed at efficiency, building a sense of ownership and capacity building for purpose of sustainability [16–18].

CLTSH is the new approach towards improving hygiene and sanitation practice and infrastructure. It emphasizes changing sanitation and hygiene behavior of communities towards open defecation free environment, hand washing practice, and keeping drinking water safe by enabling them lead their own development through issue identification and exploration, identification of action points, resource mapping, implementation of planned activities, review of progress made, and sharing of outcomes. Nowadays, it is the only approach or strategy that our country is working with [19].

This study shows that, in 314 (45.5%) respondents' village, CLTSH had been practiced. A report from tools for development stated that toilet coverage in rural villages increased from 58% to 92% in only a few months. We have to think of that it is the only approach or strategy that our country is working with [20].

ODF refers to an environment where in no feces is openly exposed to the air. It describes a state in which all community members practice use of latrine at all times and a situation wherein no open defecation is practiced at all. ODF is a term used in CLTSH to describe the attainment of 100% latrine coverage and use by all families in a community, including small children. Before a village declares ODF, it undergoes the whole steps and procedures of CLTS. So, this stage plays an important contribution for the sustainability of WASH facilities. Of the total respondents, 272 (39.4%) villagers had declared ODF. We can see that the report of ODF is less than the practiced CLTSH (39.4% and 45.5%) showing how hard it is to declare ODF. CLTSH and ODF are tools to bring sustainability of WASH facilities by promoting healthy behavior that are appropriate to the context. 440 (63.8%) of HHs had health institutions in/near their village, whereas 249 (36.2%) of HHs had no health institutions in/near their village. It is obvious that the existence of any facility nearby the community deliver services much better than those which are apart [21–23].

### 4.1. Limitation of the Study

Since the study is cross sectional, it does not show cause and effect relationship between dependent and independent variables.

### 4.2. Recommendations

Based on the findings of the study, the following recommendations were forwarded.

### 4.3. For Woreda Health Office and Woreda Water Office

- Considering WASH facilities distance from the community
- Considering community participation in the construction of WASH facilities

### 4.4. For Regional and National Policy Makers

- Promoting CLTSH and ODF strategies or tools

## 5. Conclusion

The practice of sustainability of one WASH facilities was revealed to be relatively low which is 46%. Distance from water source, community participation, practice of CLTSH in the village, village declared ODF, and existence of health institution are significantly associated with sustainability of WASH facility.

## Figures and Tables

**Table 1:** Bivariate and multivariate analysis of factors associated with sustainability of one WASH facilities in the rural settings of North Shoa, Ethiopia, November-December 30, 2020.

Variables	Sustainability of WASH facility		Crude OR (95% CI)	Adjusted OR (95% CI)
	Yes	No		
Distance from the water source				
>1.5 km	180 (76%)	56 (23.8%)	1	1
≤1.5 Km	192 (63.6%)	261 (36.4%)	4.36 (3.07–6.22)	6.46 (4.11–10.14)^*∗∗*^
Community participation				
Yes	286 (47.7%)	313 (52.3%)	23.53	29.43 (9.49–91.26)^*∗∗*^
No	86 (95.5%)	4 (4.5%)	1	1
Practice of CLTSH				
Yes	118 (37.5%)	196 (62.5%)	2.54	3.47 (2.29–5.24)^*∗∗*^
No	254 (31.0%)	121 (61.0%)	1	1
Weather the village was declared or not ODF				
Yes	121 (44.4%)	151 (55.6%)	1.38	1.62 (1.1–2.5)^*∗∗*^
No	251 (60.2%)	166 (39.8%)	1	
Existence of health institution in the village				
Yes	61 (24.4%)	188 (75.6%)	7.43	8.08 (4.75–13.74)^*∗∗*^
No	311 (70.6%)	129 (23.4%)		1

^*∗*^, *p* value ≤0.05 and ^*∗∗*^, *p* value <0.001.

## Data Availability

The data collected for this study can be obtained from the authors upon reasonable request.

## Abbreviations

CLTSH: Community Lead Total Sanitation and Hygiene
ODF: Open defecation free
WHO: World Health Organization.
